# Repeated high doses of avermectins cause prolonged sterilisation, but do not kill, *Onchocerca ochengi *adult worms in African cattle

**DOI:** 10.1186/1475-2883-4-8

**Published:** 2005-08-08

**Authors:** Barend M deC Bronsvoort, Alfons Renz, Virginia Tchakouté, Vincent N Tanya, David Ekale, Alexander J Trees

**Affiliations:** 1 Veterinary Parasitology, Division of Parasite and Vector Biology, Liverpool School of Tropical Medicine/Faculty of Veterinary, Science, University of Liverpool, Pembroke Place Liverpool,UK; 2 Fachgebiet Parasitologie, Universität Hohenheim, Germany; 3 Institut de Récherches Agricole pour le Développement, Wakwa, B.P. 65 Ngaoundere Cameroon; 4 Centre for Tropical veterinary Medicine, Royal (Dick) School of Veterinary Studies, University of Edinburgh, Easter Bush Veterinary Centre, Roslin, Midlothian, EH25 9RG, UK; 5 Universität Tübingen, Friedhofstrasse 73, 72074 Tübingen, Germany; 6 Institute of Agricultural Research for Development, Regional Centre of Bambui, B.P. 51 or 80, Bamenda, Cameroon

## Abstract

**Background:**

Ivermectin (Mectizan™, Merck and CO. Inc.) is being widely used in the control of human onchocerciasis (*Onchoverca volvulus*) because of its potent effect on microfilariae. Human studies have suggested that, at the standard dose of 150 μg/kg an annual treatment schedule of ivermectin reversibly interferes with female worm fertility but is not macrofilaricidal. Because of the importance of determining whether ivermectin could be macrofilaricidal, the efficacy of high and prolonged doses of ivermectin and a related avermectin, doramectin, were investigated in cattle infected with *O. ochengi*.

**Methods:**

Drugs with potential macrofilaricidal activity, were screened for the treatment of human onchocerciasis, using natural infections of *O. ochengi *in African cattle. Three groups of 3 cows were either treated at monthly intervals (7 treatments) with ivermectin (Ivomec^®^, Merck and Co. Inc.) at 500 μg/kg or doramectin (Dectamax^®^, Pfizer) at 500 μg/kg or not treated as controls. Intradermal nodules were removed at 6 monthly intervals and adult worms were examined for signs of drug activity.

**Results:**

There was no significant decline in nodule diameter, the motility of male and female worms, nor in male and female viability as determined by the ability to reduce tetrazolium, compared with controls, at any time up to 24 months from the start of treatments (mpt). Embryogenesis, however, was abrogated by treatment, which was seen as an accumulation of dead and dying intra-uterine microfilariae (mf) persisting for up to 18 mpt. Skin mf densities in treated animals had fallen to zero by <3 mpt, but by 18 mpt small numbers of mf were found in the skin of some treated animals and a few female worms were starting to produce multi-cellular embryonic stages. Follow-up of the doramectin treated group at 36 mpt showed that mf densities had still only regained a small proportion of their pre-treatment levels.

**Conclusion:**

These results have important implications for onchocerciasis control in the field. They suggest that ivermectin given at repeated high does may sterilise *O. volvulus *female worms for prolonged periods but is unlikely to kill them. This supports the view that control programmes may need to continue treatments with ivermectin for a period of decades and highlights the need to urgently identify new marcofiliaricidal compounds.

## Background

An estimated 17.5 million people in Africa and a further 140,000 in the Americas are infected by the filarial parasite *Onchocerca volvulus*, the cause of human river blindness [1]. The disease and its vector, *Simulium damnosum s.l*., have been the target of the largest successful and sustained vector-control programme the world has known, the Onchocerciasis Control Programme in West Africa (OCP) [2]. However in 1987 the veterinary endectocide, ivermectin, was registered as a safe and effective microfilaricide for human use against *O. volvulus *as an oral preparation, Mectizan™. The manufacturers, Merck and Co. Inc., via their Mectizan™ Donation Programme are committed to provide ivermectin free of charge to the port of entry of endemic countries 'for as long as is necessary' to aid the control of onchocerciasis. The African Programme for Onchocerciasis Control (APOC) [3] utilises ivermectin as its main tool to eliminate onchocerciasis as a public health problem. It aims to distribute ivermectin once a year through a sustainable, community-directed distribution system (CDTS).

The macrocyclic lactones are effective against a wide range of ecto- and endoparasites of animals and man. They are grouped into two major chemical families: the avermectins (including ivermectin and doramectin) and the milbemycins (including moxidectin). Ivermectin is derived from the actinomycete, *Streptomyces avermitilis*, and was originally developed as a veterinary endectocide against endo- and ectoparasites. Doramectin is a fermentation product from a mutant *S. avermectilis *strain. Its spectrum of activity is nearly the same as ivermectin [4] but its lipophilic cyclohexyl moiety at the 25-position produces a longer tissue half-life of 89 hours [5].

The efficacy of ivermectin against the skin dwelling larval stage of *O. volvulus *which causes blindness and skin lesions is well established [6-8]. However, its action against adult male and female worms is the subject of debate. Human studies with *O. volvulus *using a single dose at 150 μg/kg [9] produced no increased mortality of the adult worms and reversible affects on their fertility. Repeated 6-monthly [10-13] or 3-monthly treatments [14] produced conflicting results, but a proportion of female worms recovered their fertility 12–18 months after the last treatment and mf loads were 6–7% of pre-treatment loads 12 months after the last treatment, rising to between 9–32% at 18 months [15]. Epidemiological analysis and mathematical simulations of annual ivermectin treatments in man suggest that there is approximately a 30% reduction in mf production [16]. These authors, however, were not able to differentiate between the possible biological mechanisms such as a general loss of fecundity, or death of a proportion of the adult worms, or ivermectin facilitated immunity.

In this paper, we describe a controlled therapeutic trial in which the effects of high, repeated doses of ivermectin or doramectin were investigated against the African cattle parasite *O. ochengi *for up to 36 months after the first treatment. This species is phylogenetically closely related to *O. volvulus *for which it provides an excellent analogue [17] and was formerly adopted by the World Health Organisation (WHO) tertiary drug screening system for macrofilaricides until 2000. Previous studies have indicated both its practical merits and its predictive value [18-20].

## Materials and methods

Between February and March 1994, nine 4–6 year old Gudali cows (*Bos indicus*), each with more than 20 palpable *O. ochengi *nodules in the ventral abdominal skin, were purchased from local markets in the Adamoua province of Cameroon. They were kept at pasture on the research station of the Institut de Récherches Agricole pour le Développement (IRAD) (formerly the Institit de Récherches Zootechniques et Vétérinaires), Wakwa, 10 km S.W. of Ngaoundere, at an altitude of 1000 m where the annual transmission potential (ATP, measured in L3s/person/year) for *O. ochengi *is low, around 600 L3's (Renz and Bronsvoort unpublished observations). Treatments began in October 1994, and nodulectomies and 3-monthly skin biopsies were continued for 24 mpt. In July of 1997 (36 mpt) the experiment was terminated.

### Treatment regime

The cows were cast in lateral recumbency and the nodule distribution on each side of the ventral abdomen was mapped on a standardised recording sheet to allow tracking of individual nodules. The cows (mean weight = 379 kg) were ranked in order of their number of nodules and assigned to one of 3 groups, giving an even distribution of nodules between groups (Table 2). The cows were treated with either a subcutaneous inject of 500 μg/kg ivermectin, or a subcutaneous inject of 500 μg/kg doramectin or not treated to act as controls. Treatments were repeated at monthly intervals for 7 treatments.

**Table 2:** Nodule counts (including all removed nodules at 24 mpt), diameter (means ± standard deviations) and median age scores of female worms before and 24 months after the start of treatment in cattle, following 7 monthly treatments with ivermectin 500 μg/kg subcutaneously, doramectin 500 μg/kg subcutaneously or no treatment. Nodule numbers were adjusted for nodules removed for assay. (the number of animals in each group, n = 3 except ‡ n = 2; number of nodules per group, n = 6 except * where n = 4).

Timepoint	0 mpt			24 mpt		
	count^1^	Diameter^2 ^(mm)	♀age score^3^	count	Diameter (mm)	♀age score
Control	75 ± 26	5.9 ± 0.6	1.5	61 ± 25 ‡	6.7 ± 0.7*	2*
Ivermectin	73 ± 25	7.4 ± 1.2	1	72 ± 19	6.5 ± 0.4	3
Doramectin	73 ± 35	6.9 ± 1.1	2	66 ± 36	6.9 ± 0.9	3

^1 ^Two-way ANOVA: effect of interaction of treatment and mpt p = 0.630; effect of treatment p = 0.891; effect of mpt p = 0.510

^2 ^Two-way ANOVA: effect of interaction of treatment and mpt p = 0.270;effect of treatment p = 0.403; effect of mpt p = 0.996

^3 ^Not tested statistically

### Sampling regime

At 3-monthly intervals 3 superficial skin biopsies (between 10–20 mg) per animal were taken from a shaved patch of skin on the ventral mid-line between the umbilicus and the udder. They were incubated in Roswell Park Institute Medium (RPMI) for 24 hours followed by collagenase at 37°C for 24 hours, using the protocol of Renz *et al*. [18]. The majority of mf escaped from the skin in the first 24 hours of incubation with the remaining mf found after digestion of the skin with collagenase. The geometric mean number of mf per 100 mg of skin from the three biopsies for each animal using the log+1 transformation. The mean per group was calculated in a similar way. All animals were sampled on the same morning.

*O. ochengi *nodules numbers were assessed by careful palpation of the skin of each cow. The distribution of nodules was recorded prior to treatment and about 20 nodules per cow were permanently marked with three dots around the nodule using green tattooing ink. This ensured identification of sites where nodules had been should they disappear completely following treatment. Nodule numbers were re-counted at 6 monthly intervals until 24 mpt (8/9 animals). After recording nodule distribution, four nodules per animal were removed under local anaesthetic and the wounds closed with a catgut suture and treated with Negasunt^® ^(Bayer plc) powder to control fly strike. Two nodules per cow were preserved in 10% formal saline for histological examination. The remaining 2 nodules per cow were examined the same day by the triple assay described by Renz *et al*. [18], with the following slight modifications.

### Triple assay of live female and male worms

In brief, the nodules were trimmed of any host tissue and measured along their longest axis to give the 'nodule diameter'. Each nodule was then cut open on a hollow slide in phosphate buffered saline and the worm contents carefully expressed. The male worms were teased free and placed individually in the wells of a 24 well plate with RPMI. The anterior 5–10 mm of each female worm was amputated and placed in a separate well and classed as either 1, young (transparent white, little incrustations on the cuticle); 2, mature (yellow-brownish, with incrustations); 3, old (dark brownish, strong incrustations); or 4, calcified (black and calcified).

After incubation (30 minutes at 37°C), the motility of male worms and anterior ends of female worms were scored after 30 seconds of observation on a 3 point scale (0 = no movement; 1 = weak, slow or intermittent movement; 2 = vigorous movement). Individual worm viability was then assessed by the MTT/formazan assay [18,21]. The optical density of the formazan reduction product was determined at 492 nm (OD_492_, 'Cambridge Life Instruments photo-spectrometer'). Mean OD_492 _readings for the total male worm or anterior ends of female worms were used until 18 mpt after which the mean OD_492 _/ 10 mm of worm was calculated.

The remaining (posterior) part of each female worm was then washed into a mortar with 1.8 ml of PBS (final volume 2 ml), gently crushed with a pestle and a sample transferred to a Fuchs-Rosenthal chamber (3.2 μl) to determine an embryogram following the method of Schulz-Key [22]. A count was made of the normal and abnormal/pathologic embryonic stages and intra-uterine mf. Oocytes were also counted, but the repeatability of their counts was not reliable and they were not included in the final analysis.

### Statistical Analyses

Statistical analyses were performed using 'Minitab'^1 ^and BMDP^2 ^computer packages. The nodule counts, nodule diameters and the MTT reduction/formazan formation results were analysed using a two-way repeat measures ANOVA between groups and timepoints. Worm age, motility scores and the percentage pathological stages at each timepoint were compared using a Kruskal-Wallis non-parametric one-way ANOVA. All statistical tests were interpreted at the 5% level of significance.

## Results

Of the original nine cows at the start of the study, one control animal, no. 17, had to be slaughtered after the 12 mpt sampling on welfare grounds. Two more animals, one control and one ivermectin treated animal, were removed for different reasons after the 24 mpt sampling and regular observations were terminated at this point. However, where possible skin mf loads were monitored and nodules collected from the remaining six animals until 51 mpt.

### Microfilariae

Following treatment, the mf density in the skin declined sharply and no skin mf were found at 3 mpt (Table 1). The control animals maintained substantial mf loads throughout the study, although their loads fluctuated considerably. Small numbers of mf began reappearing in 4/6 of the treated animals between 15 and 21 mpt.

**Table 1:** Geometric mean (3 skin snips)*O. ochengi *mf density+1 per 100 mg skin in cattle following 7 monthly treatments with ivermectin 500 μg/kg subcutaneously, doramectin 500 μg/kg subcutaneously or untreated controls. Animals lost to follow-up are indicated in the table.

	**ID**	0 mpt	3 mpt	6 mpt	9 mpt	12 mpt	15 mpt	18 mpt	21 mpt	24 mpt	36 mpt
Control	**17**	3803	635	203	0	18	a				
	**19**	841	260	1515	774	1135	604	1698	185	257	c
	**22**	4578	1583	911	712	767	291	982	975	1462	281

Ivermectin	**14**	509	0	0	0	0	1	3	0	8	2
	**18**	671	0	0	0	0	0	2	0	0	b
	**20**	240	0	0	0	0	0	0	0	0	0

Doramectin	**12**	6901	0	0	0	0	0	0	5	16	75
	**15**	1607	0	0	0	0	0	0	0	0	0
	**23**	146	0	0	0	0	0	3	2	0	0

a: Slaughtered following tetracycline therapy for dermatophilus infection, b: slaughtered due to septic arthritis, c lost to follow-up

### Nodule counts, diameter and female age score

Table 2 gives the mean nodule counts, the mean nodule diameter and the median female age score (male age scores were not included) for the 3 groups before and 24 months after the start of treatment. There was no significant difference in nodule counts or nodule diameters between the groups or time points.

### Motility, number of males and females per nodule and viability as measured by formazan formation

**Figure 1:**
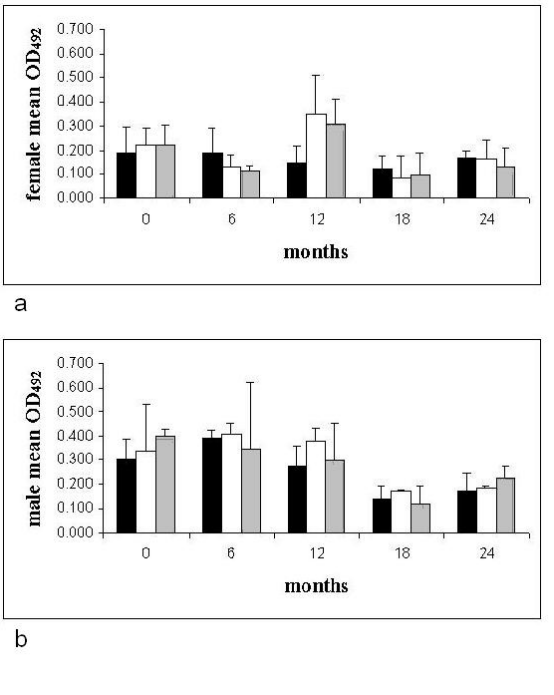
Mean (with standard deviation) OD_492 _of female (a) and male (b) *O. ochengi *formazan formation after MTT reduction before and up to 24 months after treatment (from 18 months after the start of treament OD_492 _is standardised for 10 mm of length). ■ = control, □ = ivermectin,  = doramectin. (N.B. The mean OD_492 _reading for males was first calculated for each nodule and this was then used to calculate the group means given in Figure 1b) Foot notes. (a) Two-way ANOVA for effect of timepoint and treatment group: Interaction effect between group and timepoint p = 0.44; group effect p = 0.62; effect of timepoint p = 0.02. (b) Two-way ANOVA for effect of timepoint and treatment group: Interaction effect between group and timepoint p = 0.69; group effect p = 0.79; effect of timepoint p = 0.02.

Male *Onchocerca *worms are mobile and although the number per female varied during the experiment, the ratio of males to females remained around one (with one female worm to each nodule). The male and female median motility scores are summarised in Table 3 and Figure 1. The median motility of male worms remained at 2 throughout the study except at 18 mpt for the ivermectin group. Similarly, for female anterior ends, their motility was 2 except at 24 mpt when this dropped. A Kruskal-Wallis ANOVA comparing the group medians for this timepoint was not significant (p = 0.20).

**Table 3:** The ratio of the number of male/female (♂/♀) worms examined for each group at each timepoint (one female worm was found per nodule) and the median male (♂MOT) and (♀MOT) motility score before and up to 24 months the after the start of treatment in cattle following 7 monthly treatments with ivermectin 500 μg/kg subcutaneously, doramectin 500 μg/kg subcutaneously or untreated controls.

mpt		0	6	12	18	24
Control	♂/♀	5/6	5/5	8/5	4/4	3/4
	♂MOT	2	2	2	2	2
	♀MOT	2	2	2	2/6	2
Ivermectin	♂/♀	3/6	5/6	6/6	0.3	3/6
	♂MOT	2	2	2	1.5	2
	♀MOT	2	2*	2	2*	1.5*
Doramectin	♂/♀	11/6	5/6	9/6	6/6	9/6
	♂MOT	2	2	2	2	2
	♀MOT	2	2	2	2	0.5**

*= 1 calcified dead worm, **= 2 calcified dead worms

From Figures 1a and 1b there is clearly no drug effect on worm viability/OD_492 _of either males or females. There is an effect of time with mean OD_492 _readings dropping after 18 mpt. This effect is associated with the change to a standardisation of the viability/OD_492 _per 10 mm of worm length.

## Embryograms

**Figure 2:**
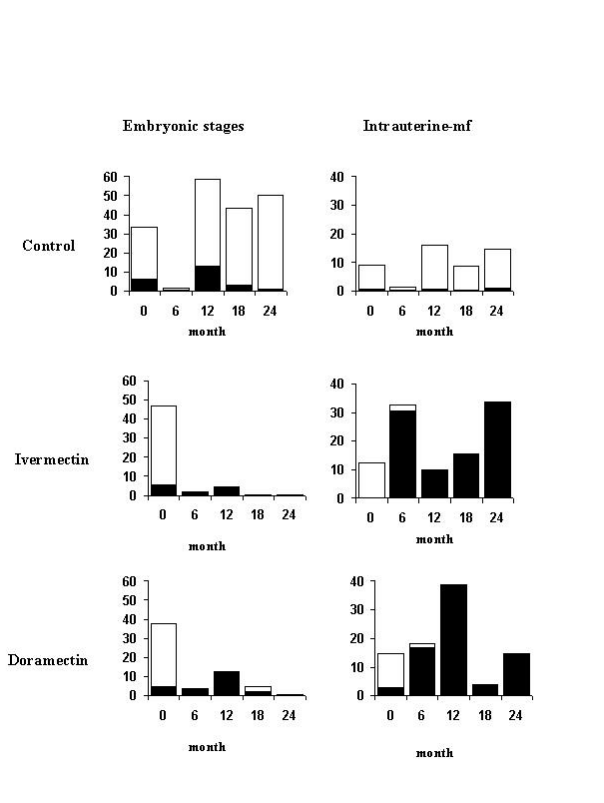
Mean number (× 1000) of embryonic stages 'o' (small and large morulae, horseshoe and pretzel stages) and intra-uterine mf of *O. ochengi *per nodule, over-laid with the number of pathological stages '■ 'calculated from the median percent of pathological forms before and up to 24 months following treatment with ivermectin or doramectin.

The results of the embryograms are given in Figure 2. Normal embryonic stages (small morulae, large morulae, horseshoes, pretzels or coiled microfilariae) disappeared from the two treated groups by 6 mpt and failed to reappear in the female worms. Intrauterine mf accumulated and finally degenerated in the uteri, failing to be expelled for more than 24 months from the start of treatment. Beginning with approximately 10,000 normal intra-uterine mf stages pre-treatment, the number of pathological stages peaked around 30–40,000 at 6 to 12 mpt. The intra-uterine mf disintegrated steadily, first becoming vacuolated and non-motile, and eventually becoming ghost forms. This sterilizing effect persisted in 4 of the 6 treated animals for up to 24 months from the start of treatment. In two of the doramectin treated animals a nodule (i.e. 2/6 nodules), classed as old, was recovered at both the 18 and 24 mpt examinations, with large numbers of normal embryonic and intra-uterine mf amongst the pathological stages still in the uteri. In the ivermectin-treated animals, no fertile nodules were found, but the small number of mf in the skin in one animal (no. 14) indicates the presence of at least one fertile worm couple.

## Discussion

Previous drug trials [18,23] have demonstrated the advantages of the *O. ochengi *screen in cattle in which individual animals are sequentially sampled and worm viability is determined.

As in previous studies in cattle [18], the skin mf density declined to zero following treatment with ivermectin and the same result was found with doramectin. Although mf began reappearing around 18 mpt only one animal, no. 12, showed a consistent increase in mf density and therefore the presence of at least one fertile worm couple. An interesting observation was the disappearance of mf from the skin of a control animal, no. 17. This was associated with repeated treatments of oxytetracyclines for a severe mycotic drematitis (*Dermatophilus congolensis*) skin infection. Treatment failed to control the skin infection and the animal was culled on welfare grounds after the 12-mpt sampling. The macrofilaricidal effect of oxytetracyclines was subsequently demonstrated [23].

In spite of being given at 2.5 times the recommended dose for cattle and at monthly intervals for 7 treatments, neither ivermectin nor doramectin reduced nodule diameter, male or female worm number or male motility compared with controls. Female motility appeared to be compromised in the doramectin treated group by 24 mpt, though this was not statistically significant. The viability/OD_492 _of female worms, however, did not appear to have been reduced significantly. Taken together these results support the conclusions of previous studies in humans taking invermectin once or twice annually that even using high and prolonged dosing, the avermectins are not macrofilaricidal against *Onchocerca spp*. [10,12,13]. The failure to kill the adult worm is, however, not due to a failure of the drug to penetrate the *O. ochengi *intra-dermal nodule [24,25].

The main effect of both ivermectin and doramectin on the adult female worm was on the development of the multicellular embryonic stages and the intra-uterine mf, as in man [10,12,13] where repeated treatments of ivermectin at 150 μg/kg every 6–12 months were reversible after 9–18 months though fecundity had been reduced. Interestingly, the number of intra-uterine mf rose sharply in both treated groups as dead and dying mf accumulated in the uteri of the worms. Over the same period the embryonic stages disappeared from the uteri. Certainly some of these must have continued to develop through to mf in order to explain these changes [26]. Why the degradation and disappearance of dead mf from the uteri is so protracted, is unclear, but it may be important in preventing fertilisation of the female [11,27], as this is the stimulation for a new cycle of oogenesis [28]. Alternatively, the uteri may be paralysed as happens to the pharyneal muscles of *Haemonchus contortus *[29].

Observations in man noted a disappearance of *O. volvulus *males from nodules following multiple treatments with ivermectin [14,30]. In addition, after treatment with other macrofilaricidal drugs, fewer males were recovered from *O. ochengi *nodules [18] and this is considered a useful, early indicator of macrofilaricidal activity. Our study, however, is in agreement with observations on *O. volvulus *in man [11,12] as we found male numbers to vary over time with no clear decline. Further, the male worms maintained their motility and viability (OD_492_) throughout the 24 months of the study. The effects of avermectins on male fertility were not examined here.

Our study has demonstrated that with higher doses of ivermectin or doramectin, repeated at monthly intervals, embryogenesis can be disrupted and possibly permanently impaired. Even 18 months after the last treatment none of the removed ivermectin treated nodules showed signs of recommencing embryogenesis. At 24 mpt, 2/6 nodules in the doramectin group did appear to regain some reproductive capability.

In previous studies of age-specific nodule counts [31] it has been suggested that nodules continue to be acquired throughout the life of the cow while their mf loads level off around 4–6 years of age. From the prophylactic studies in cattle [19,32] it is clear that ivermectin and moxidectin can prevent the development of L3 larvae in the host for at least 3 months after a single treatment. The corrals at Wakwa have a known low ATP of around 600 L3's per animal per year as opposed to 24,000 L3's per year near the *Simulium *breeding sites (Renz and Bronsvoort unpublished observations). In our study although there was a low level of transmission in the area, the nodule burden of the animals did not alter significantly up until 24 mpt after allowing for the removal of 20 nodules per animal. An alternative hypothesis could be that there is some form of density-dependent regulation of the adult worms with only very low turnover of the adult population. This theory is supported by the observation that control animals in the current experiment failed to acquire new nodules in the first year as determined by the regular 6 monthly counts. In addition different trials where L3's were experimentally inoculated into calves have failed to produce a clear positive correlation between the number of infective larvae inoculated and the number of nodules that subsequently developed [33,34]. If in fact there is some form of density dependent regulation of the adult population with individual worm burdens determined by some host-parasite balance then it may actually be more beneficial to sterilise the female worm, thus preventing the majority of new infections from developing.

The sterilising effect of high doses of ivermectin, combined with their prophylactic potential [19], will ensure the usefulness of this drug and/or related compounds. With community-directed distributions being set up under the APOC scheme there will be a much improved potential for timing treatments and/or increasing the frequency of treatments to maximise these other useful effects. High doses and increased frequency of treatment has been shown here to reduce female worm fertility in *O. ochengi *and similar results have been demonstrated in humans for *O. volvulus *[35]. This potentially could help to reduce the length of control programmes though it is doubtful whether high coverage levels could be sustained long enough to achieve worldwide eradication [36]. In addition, work on *O. ochengi *suggests that people receiving ivermectin are unlikely to develop immunity to *O. volvulus *and are therefore highly susceptible should drug distribution cease [32]. However, WHO itself recognises that ivermectin by itself can not completely interrupt transmission of *O. volvulus *[37-39] and therefore work should continue to find and develop an effective macrofilaricide that would be used in combination with ivermectin.

In conclusion, although this experiment has failed to demonstrate that high and repeated doses of ivermectin or doramectin are macrofilaricidal, it has shown that they both have a profound effect on embryogenesis producing a prolonged period of sterility in female worms. However, simulation modelling suggests that ivermectin alone will not interrupt transmission [36]. This appears to be born out by epidemiological studies from the field where the conclusion was that repeated ivermectin mass annual treatment will not lead to the elimination of transmission of onchocerciasis from West Africa though data on 6-monthly treatments was not sufficient to draw a conclusion [39]. There is, therefore, still an urgent need to continue the search for a safe effective macrofilaricidal compound.

## Authors' contributions

**BB: **Carried out the field work and project management in Cameroon and was responsible for data analysis and preparation of the manuscript

**AR: **Responsible for project inception and design and preparation of the manuscript

**VNT: **Responsible for field work, project set-up and design and overall project coordination in Cameroon

**VT: **Responsible for field work and project set-up

**DE: **Responsible for analysis and interpretation of field samples in the laboratory

**AT: **Responsible for project inception and design and preparation of the manuscript

## Note

^1^Minitab Inc, http:\\www.minitab.com

^2^BMDP Statistical Software, Inc., Cork Technology Park, Cork, Ireland.
